# Proteomic analysis of signaling pathways modulated by FABP5 in macrophages

**DOI:** 10.21203/rs.3.rs-3332029/v1

**Published:** 2023-09-12

**Authors:** Faniya Doswell, John D. Haley, Martin Kaczocha

**Affiliations:** Molecular and Cellular Biology Program, Stony Brook University, Stony Brook, NY, USA; Department of Anesthesiology, Renaissance School of Medicine, Stony Brook University, Stony Brook, NY, USA; Department of Pathology, Renaissance School of Medicine, Stony Brook University, Stony Brook, NY, USA; Stony Brook Biological Mass Spectrometry Facility, Renaissance School of Medicine, Stony Brook University, Stony Brook, USA; Department of Anesthesiology, Renaissance School of Medicine, Stony Brook University, Stony Brook, NY, USA

**Keywords:** Inflammation, FABP5, fatty acid binding protein 5, macrophage, polarization, proteomics, phosphoproteomics

## Abstract

**Background:**

While acute inflammation serves essential functions in maintaining tissue homeostasis, chronic inflammation is causally linked to many diseases. Macrophages are a major cell-type that orchestrates inflammatory processes. During inflammation, macrophages undergo polarization and activation, thereby mobilizing pro-inflammatory and anti-inflammatory transcriptional programs that regulate ensuing macrophage functions. Fatty acid binding protein 5 (FABP5) is a lipid chaperone that is highly expressed in macrophages. FABP5 deletion is implicated in driving macrophages towards an anti-inflammatory phenotype, yet the signaling pathways regulated by macrophage FABP5 have not been systematically profiled. Herein, we leveraged proteomic and phosphoproteomic approaches to characterize pathways modulated by FABP5 in M1 and M2 polarized bone marrow derived macrophages (BMDMs).

**Results:**

Stable isotope labeling by amino acids (SILAC) based analysis of M1 and M2 polarized wild-type (WT) and FABP5 knockout (KO) BMDMs revealed numerous differentially regulated proteins and phosphoproteins. FABP5 deletion impacted several downstream pathways associated with inflammation, cytokine production, oxidative stress, and kinase activity. Kinase enrichment analysis based on phosphorylated sites revealed key kinases, including members of the GRK family, that were altered in FABP5 KO BMDMs. Reactive oxygen species (ROS) levels were elevated in M1 polarized KO macrophages, consistent with the differential protein expression profiles.

**Conclusions:**

This study represents a comprehensive characterization of the impact of FABP5 deletion upon the proteomic and phosphoproteomic landscape of M1 and M2 polarized BMDMs. Loss of FABP5 altered multiple pathways implicated in inflammatory responses and macrophage function. This work provides a foundation for future studies seeking to investigate the therapeutic potential of FABP5 inhibition in pathophysiological states resulting from dysregulated inflammatory signaling.

## Introduction

Acute inflammation represents an important protective mechanism in response to potentially harmful and foreign pathogens, which is followed by resolution of inflammation and return to homeostasis (1, 2). In contrast, chronic unresolving inflammation predisposes to a myriad of diseases that account for over 50% of deaths globally (2, 3). Inflammation triggers the activation of innate immune cells including macrophages, resulting in the transcriptional activation of inflammatory pathways, release of a variety of inflammatory mediators, and recruitment of additional immune cells (1, 4). Macrophages are highly plastic cells and quickly respond to a changing microenvironment. Macrophages are commonly categorized based on their function: M1-like macrophages release pro-inflammatory cytokines while M2-like macrophages release anti-inflammatory cytokines (5, 6). Classically activated M1 macrophages are polarized by lipopolysaccharide (LPS) and T-helper 1 (Th1) cytokines such as interferon-γ (IFNγ) (7, 8). M2 phenotypes can be further divided into M2 subcategories that include M2a, M2b, M2c, and M2d (7). In general, M2 macrophages are known to be polarized by T-helper 2 (Th2) cytokines that have different anti-inflammatory effects which are responsible for the functional differences comprising the M2 subclasses (7).

Fatty acid binding proteins (FABPs) are a family of cytoplasmic proteins with specialized functions in lipid transport, metabolism, and signal transduction (9). FABP5 is a prominent FABP that is highly expressed in macrophages and is thought to modulate inflammation through diverse mechanisms including the regulation of lipid metabolism and nuclear receptor signaling (9). Previous studies suggest that FABP5 regulates macrophage responses *in vitro* and *in vivo*. The loss of FABP5 confers an anti-inflammatory phenotype in a liver injury model (10). Similarly, deletion of FABP5 blunts the inflammatory upregulation of microsomal prostaglandin E synthase 1 that mediates prostaglandin E2 biosynthesis (11). FABP5 regulates anti-inflammatory responses in lung macrophages via peroxisome proliferators activated receptor γ (PPARγ) *in vivo* (12, 13). Prior studies demonstrate that bone marrow derived macrophages (BMDMs) obtained from FABP5 KO mice express higher levels of anti-inflammatory mediators following LPS and IFNγ stimulation (10). Moreover, the absence of FABP5 in BMDMs elevates the expression of anti-inflammatory markers after M2 polarization with interleukin-4 (IL-4) (10). Recent studies by Hou et al. revealed that FABP5 deletion decreases pro-inflammatory cytokines in LPS-stimulated macrophages and enhances M2 polarization following IL-4 stimulation (14, 15).

Regulation of inflammatory responses by FABP5 in polarized macrophages has been largely investigated at the RNA level (10, 14). Given the emerging functions of FABP5 in macrophage biology and the therapeutic potential of targeting FABP5 to treat inflammatory diseases, the goal of this study was to systematically characterize proteins and signaling pathways modulated by macrophage FABP5. Combining stable isotope labelling with amino acids in cell culture (SILAC) and phosphoproteomic approaches, we report on the global landscape of WT and FABP5 KO macrophages, including differential protein expression and pathway engagement in M1 and M2 polarized BMDMs.

## Methods

### Animals

The experiments were approved by Stony Brook University Institutional Animal Care and Use Committee (#277150). Adult male and female WT C57Bl/6 and FABP5 KO mice were kept on a 12:12-h light:dark cycle with *ad libitum* access to food and water. The mice were euthanized with isoflurane and BMDMs were isolated as described below.

### Cell culture and SILAC labeling

BMDMs were obtained from the femur and tibia of 8 WT and 8 FABP5 KO mice as described previously (16, 17). The femur and tibia were cleaned with gauze and the ends of each bone removed. The bones were flushed with 1X DPBS (Gibco) to collect bone marrow in a sterile Falcon tube. The bone marrow samples were then processed with red blood cell lysis buffer Hybri-Max (Sigma, Cat. # R7757–100ML). Reagents from SILAC protein quantitation kit (ThermoFisher Scientific, Cat. # A33970) were used for cell culture media. Additionally, ^13^C_6_^15^N_4_ L-Arginine-HCl was also used to supplement the BMDM SILAC media (ThermoFisher Scientific Cat. # 899990). The cells were incubated in open DMEM-F12 for 4 hours at 37°C and supernatant was collected from petri dishes to eliminate resident cells. The cells were incubated at 37°C, 5% CO_2_ with SILAC-BMDM medium containing 15% L-Cell Conditioned Media (LCM), 10% dialyzed FBS and 1% penicillin/streptomycin (Gibco) in DMEM-F12. SILAC media contained DMEM:F12, dialyzed fetal bovine serum (FBS), and either “light” (^12^C-K and -R) or “heavy” (^13^C_6_-K, ^13^C_6_-^15^N_4_-R) lysine and arginine. WT BMDMs were incubated in “light” while FABP5 KO BMDMs were incubated in “heavy” media at 37°C, 5% CO_2_ for 7 days.

### Polarization of macrophages

BMDMs were polarized into the M1-like condition by incubating the cells with 100 ng/mL LPS and 20 ng/mL IFNγ for 24 h. M2-like macrophages were polarized using 20 ng/mL IL-4 for 24 h. Unstimulated macrophages were given media replacements without any additional stimulatory agents. Following amino acid labeling, each polarized condition had a corresponding non-polarized condition that was run in parallel.

### Cell Harvesting

Macrophages were washed with 1X DPBS. Cells were then harvested by scraping in lysis buffer containing 50 mM triethylammonium bicarbonate in 5% SDS supplemented with PhosSTOP phosphatase inhibitor tablet. Heavy and light SILAC labeled samples were combined and processed for protein and phosphopeptide analyses.

### Total protein analysis preparation

For total protein analysis, 100 μL of cell lysates were reduced in 10 mM DTT at 55°C for 30 min, followed by alkylation in 25 mM iodoacetamide at room temperature in the dark for 30 min. Then, 10 μL of 12% phosphoric acid was added to the samples, followed by 7000 μL of S-Trap bind/wash buffer (90% methanol/50 mM TEAB) to produce a micro precipitate. The samples were then loaded on an S-Trap mini cartridge (cat. # K02-mini-10 Protifi), washed three times with S-Trap bind/wash buffer, followed by centrifugation at 4000 × g for 1 min. The samples were digested with trypsin (20 μg) in 50 mM TEAB in a humidified incubator overnight at 37°C. Peptides were eluted by sequential addition of 80 μL of 50 mM TEAB, 0.2% formic acid, and 50% acetonitrile, 0.2% formic acid, each followed by centrifugation at 4000 × g for 1 min. The samples were then dried by SpeedVac and resuspended in 200 μL of 0.1% trifluoroacetic acid (TFA) for desalting on HLB reverse phase cartridges (Waters) and eluted to generate 20% and 50% acetonitrile fractions.

### Phospho-peptide analysis preparation

For phospho-peptide analysis, SILAC heavy- and light-labeled proteins were combined and precipitated as above in 1.1% phosphoric acid, 90% methanol/100 mM TEAB. The protein precipitates were centrifuged at 2000 × g for 10 minutes, transferred to a fresh microfuge tube, and washed five times with 90% methanol/50 mM TEAB. One milligram sample was digested with trypsin (100 μg) in 50 mM TEAB in a humidified incubator overnight at 37°C. The peptides were acidified with 1% TFA, desalted on HLB reverse-phase cartridges with 50% ACN. The peptides were dried, and phospho-peptides were isolated by TiO_2_ affinity chromatography (10 μm beads; glsciences.com). The dried peptides were resuspended in 500 μL 0.1% TFA, 50% acetonitrile (ACN), and 1M lactic acid. Peptide samples were added using 30 mg TiO_2_ beads and mixed at 1000 rpm (Eppendorf Thermo mixer) at room temp for 90 min. The TiO_2_ beads were washed three times with 0.1% TFA, 50% acetonitrile (ACN). Phosphopeptides were eluted with 50 mM KH_2_PO_4_ pH 10.5 (pH adjusted with NH_4_OH), immediately neutralized with 5% formic acid, 50% acetonitrile, and lyophilized. Peptide fractions were desalted using HLB cartridges and eluted to generate 20% ACN and 50% ACN fractions. Dried phosphopeptides were resuspended in 0.1% FA and subjected to LC-MS/MS.

### Mass spectrometry

Parent peptide mass, collision-induced fragment mass information, and peptide abundance values were obtained by liquid chromatography-electrospray ionization tandem mass spectrometry (LC-MS/MS) using an orbitrap instrument (Thermo Q-Exactive HF), followed by protein database searching. HPLC C18 columns were prepared using a P-2000 CO_2_ laser puller (Sutter Instruments) and silica tubing (100 μm ID × ~20 cm), and they were self-packed with 3 μm Reprosil C18 resin. Peptides (~ 10 μg in a 1 μL injection volume) were separated using both 120-minute and 180-minute gradients. Gradient 1 (120 minutes) used a flow rate of 300 nL/min, with a gradient elution step of 0–40% acetonitrile (ACN) and 0.1% formic acid (0.23%/min) over 90utes, followed by a 40–60% ACN gradient over 15 minutes and a 10-minute wash with 90% ACN. Gradient 2 (180 minutes) was similar but used a 0–10% ACN gradient over 15 min, 10–45% over 140 min, 45–60% over 12 min, and 60–90% over 5 min.

### Data processing and statistical analysis

Peptide identification and quantitation were performed using an orbital trap (Q-Exactive HF; Thermo) instrument, followed by protein database searching using Proteome Discoverer 2.4. Four technical replicates per sample were analyzed: both 20% ACN and 50% ACN fractions for eight LC-MS/MS runs per sample in total. Electrospray ionization was achieved using a spray voltage of ~ 2.2 kV. Information-dependent MS acquisitions were made using a survey scan covering m/z 375–1400 at 60,000 resolutions, followed by ‘top 20’ consecutive second product ion scans at 15,000 resolution. AGC targets for MS and MS/MS were 5e5 and 5e4, with a maximum IT of 100 ms and 50 ms, an MS/MS loop size of 20, and dynamic exclusion for 15 s. Mass resolution cutoffs for MS and MS/MS were 10 ppm and 0.05 Da, respectively. Data files were acquired with Xcalibur. Peptide alignments and quantitation were performed using Proteome Discoverer v2.4 software (Thermo). Protein false discovery rates experiments were binned at 0.01 and 0.05 FDR. Peptide and PSM FDR cutoffs were typically set to 0.01. Two missed tryptic cleavages were allowed, and modifications considered included static cysteine derivatization, and variable deamidation (NQ), water loss (ST), oxidation (M), phosphorylation (STY), and SILAC labels. Pairwise peptide ratios between heavy and light SILAC-labeled samples allowed t-test calculations based on the background population of peptides. The human UniProt dataset (73,101 entries) was used for data alignment. Fold change ratios of FABP5 KO (heavy) and WT (light) for each condition was obtained by matched peptide-based label-free quantitation, and p-values were calculated by Benjamini-Hochberg correction for FDR. Coefficients of variation between biological and technical replicates were used to measure subject variability and quality control, respectively. The data were obtained from eight biological and two technical replicates.

### Proteomics and phosphoproteomics bioinformatics analysis

For non-SILAC proteomic analysis, grouped abundances of WT and KO were used separately to depict changes in expression at the protein level for each condition. Any blank values, excluding Arg1, were excluded from the list. SILAC provides further quantitative capabilities for proteomic and phosphoproteomic changes. This allows for changes to be quantified between the FABP5 KO and WT BMDMs. Thus, abundance ratios were quantified as FABP5 KO/WT (heavy/light). Proteins and phosphorylated proteins were considered statistically significant if log_2_ fold change (log_2_FC) > 1 and < −1 with a threshold of FDR adjusted p-value < 0.05. These differentially expressed proteins and phosphoproteins were depicted in a volcano plot, using R EnhancedVolcano package in Rstudio (18). The entire set of related proteins and phosphoproteins were used to perform gene set enrichment analysis (GSEA). The pathway enrichment analyses were performed using the clusterProfiler R package with gene ontology (GO) and Kyoto Encyclopedia of Genes and Genomes (KEGG) databases to associate the proteins and phosphoproteins with their functions and pathways (19, 20). GO analysis was performed through the gseGO function and KEGG analysis was performed with the gseKEGG function. The proteins and phosphoproteins were ranked based on log_2_ fold-change logarithmic values and adjusted p-value threshold of 0.05. Kinase enrichment analysis was performed with PhosphoSitePlus database and Maayan Lab Kinase Enrichment Analysis (KEA) (21, 22). The phosphorylation sites in correspondence to the FABP5 KO/WT phosphoproteins were used for PhosphoSitePlus analysis. These FABP5 KO/WT phosphopeptides were analyzed for kinase enrichment with a log_2_ fold change threshold of 1 and p-value threshold of 0.05. The KEA kinase network analysis was performed using an input list of FABP5 KO/WT proteins that had phosphorylated sites with log_2_ fold change threshold of |0.8|.

### M1 and M2 qPCR Validation

RNA was extracted from WT and FABP5 BMDM samples, then cDNA synthesis was performed using SuperScript III First Strand Synthesis (ThermoFisher). qPCR was performed using PowerUp SYBR green (ThermoFisher) on a StepOnePlus instrumentation (Applied Biosystems). The following primers were used: β-actin, forward 5’-GACGGCCAGGTCATCACTAT-3’and reverse 5’-CGGATGTCAACGTCACACTT-3’; IL-1β, forward 5’- GCTTCAGGCAGGCAGTATC – 3’ and reverse 5’- AGGATGGGCTCTTCTTCAAAG – 3’; COX-2, forward 5’-AGGACTGGGCCATGGAGT-3’and reverse 5’-ACCTCTCCACCAATGACCTG-3’; TNFα, forward 5’-GAACTGGCAGAAGAGGCACT-3’ and reverse 5’-AGGGTCTGGGCCATAGAACT-3’; Arg-1, forward 5’-CTCCAAGCCAAAGTCCTTAGAG-3’ and reverse 5’-AGGAGCTGTCATTAGGGACATC-3’; YM-1, forward 5′-TCTGGTGAAGGAAATGCGTAAA-3 and reverse 5′-GCAGCCTTGGAATGTCTTTCTC-3′; Fizz-1, forward 5′-CAGCTGATGGTCCCAGTGAA-3′ and reverse 5′-TTCCTTGACCTTATTCTCCACGAT-3′. Quantification was performed utilizing the 2^−ΔΔCt^ approach, where β-actin served as the housekeeping gene and samples were normalized based on unstimulated WT levels.

### ROS measurement

BMDMs were seeded at 5*10^5^ cells/ plate and were labeled with 5 uM of CellROX Deep Red (Invitrogen, cat. # C10422) at 37°C for 30 min. Cells were also labeled with Zombie staining (Biolegend, cat. # 423105) to quantify viable cells. Cells were harvested and analyzed via flow cytometry by using the mean fluorescence intensity (MFI) in a logarithmic scale. Statistical analysis was performed with GraphPad using Bonferroni-Dunn unpaired t-test method.

## Results

### M1 and M2 polarization in WT and FABP5 KO BMDMs

We first compared the proteomic and phosphoproteomic landscape of unstimulated SILAC-labeled WT and FABP5 KO BMDMs. Changes between KO and WT BMDMs prior to polarization are shown in Supplemental Fig. 1A and 1B. A total of 3740 proteins were detected, with 358 downregulated and 271 upregulated in FABP5 KO BMDMs (Supplemental Fig. 1A). GO analysis revealed that positive regulation of cytokine production, response to oxidative stress, and protein kinase regulator activity pathways were differentially regulated (Supplemental Fig. 1C). KEGG analysis demonstrated that pathways related to basal transcription factor are enriched while mRNA surveillance pathway is suppressed in FABP5 KO BMDMs (Supplemental Fig. 1D). These findings suggest potential differences in the regulation of cytokines, kinases, and oxidative stress in response to loss of FABP5.

**Figure 1:**
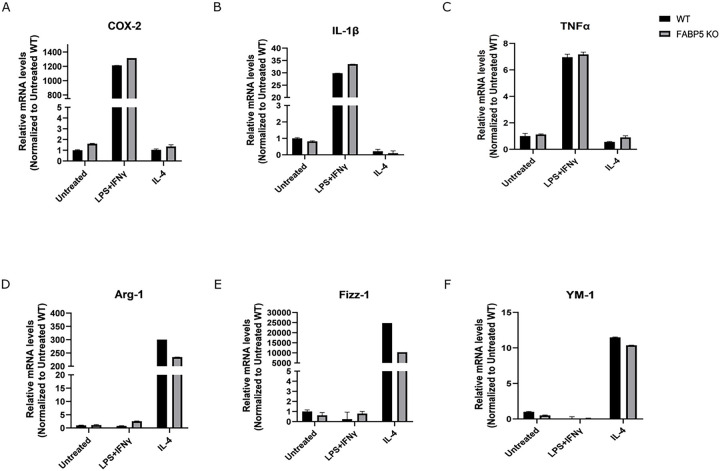
Confirmation of M1 and M2 polarization in WT and FABP5 KO BMDMs. BMDMs were treated with LPS+IFNγ or IL-4 for 24 h to induce M1 and M2 polarization, respectively. Relative expression of M1 markers COX-2 **(A)**, IL-1β **(B)**, and TNFα **(C)** as well as the M2 markers Arg-1 **(D)**, Fizz-1 **(E)**, and YM-1 **(F)** was quantified via qPCR (n = 4).

**Figure 2:**
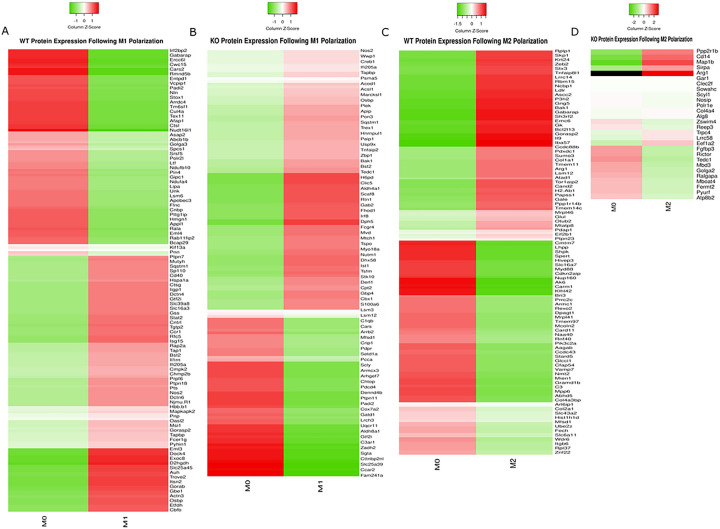
Validation of BMDM M1 and M2 polarization at the protein level. Proteomic analysis was performed in WT and FABP5 KO BMDMs by comparing protein expression between M0 vs M1 and M2 conditions. Heatmaps of up- and down-regulated proteins are displayed for WT **(A)**and FABP5 KO **(B)** BMDMs after M1 polarization stratified by Z-score. Heatmaps of up- and down-regulated proteins for WT **(C)** and FABP5 KO **(D)**BMDMs after M2 polarization.

BMDMs were polarized into an M1-like state using LPS and INFγ and M2-like state using IL-4. M1 polarization in WT and FABP5 KO BMDMs was confirmed via qPCR using the M1 canonical markers interleukin-1β (IL-1β), tumor necrosis factor α (TNFα), cyclooxygenase-2 (COX-2), and IL-6 (Fig. 1A–C). M2 polarization was confirmed using arginase-1 (Arg-1), Fizz-1 (also known as resistin-like α/Retnlα), and chitinase-like protein 3 (YM-1) (Fig. 1D–F). We next assessed proteomic changes that accompany M1 and M2 polarization in WT and FABP5 KO macrophages (Fig. 2). Pro-inflammatory mediators including Nos2 (also known as iNos), cluster of differentiation 40 (CD40), and C-C chemokine receptor 1 (CCR1) were upregulated in WT M1 BMDMs (Fig. 2A). In the M2 polarized BMDMs, Arg1 was prominent amongst the upregulated proteins (Fig. 2C), consistent with the qPCR results. Similar results were observed for the FABP5 KO samples (Fig. 2B, 2D). Overall, these data confirm that stimulation with LPS and IFNγ or IL-4 polarizes WT and FABP5 KO BMDMs to their pro- and anti-inflammatory M1-like and M2-like states, respectively.

### SILAC-based proteome and phosphoprotome profiling following M1 and M2 polarization

**Figure 3:**
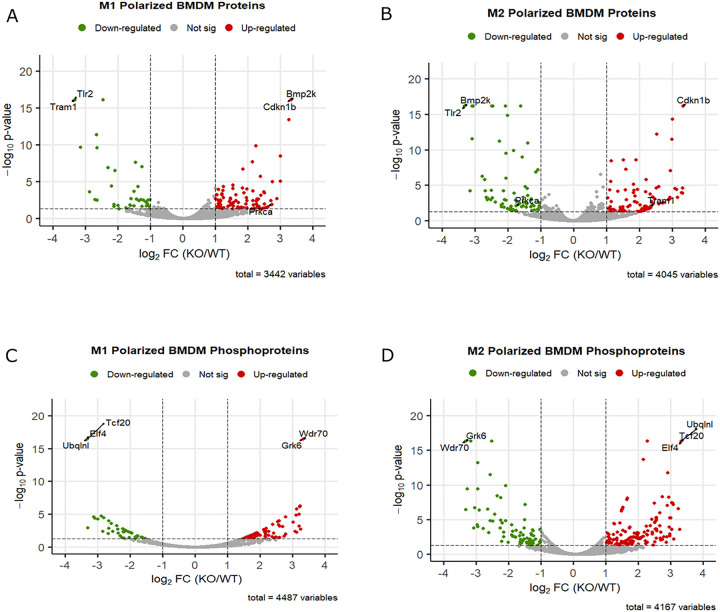
SILAC Proteomic and phosphoproteomic profile of M1 and M2 polarized WT and FABP5 KO BMDMs. Volcano plots depict differential expression of proteins and phosphoproteins between FABP5 KO and WT BMDMs presented as log_2_FC of FABP5 KO/ WT abundance ratios with the log_2_FC threshold set at 1 and −log_10_ p-value threshold set at 1.3. Differential protein expression for M1 polarized **(A)** and M2 polarized **(B)** BMDMs is shown. Differential expression of phosphoproteins for M1 **(C)** and M2 **(D)** polarized BMDMs. Downregulated proteins and phosphoproteins are shown in green. Upregulated proteins and phosphoproteins are illustrated in red.

We employed quantitative SILAC analysis to identify alterations in protein and phosphoprotein expression between polarized WT and FABP5 KO BMDMs. We observed 326 downregulated and 239 upregulated proteins in M1-polarized KO BMDMs compared to WT controls (Fig. 3A). Similarly, M2 polarized BMDMs contained 446 downregulated and 253 upregulated proteins (Fig. 3B). Phosphoproteomic analysis further revealed distinctive changes in phosphorylation events with 335 downregulated and 203 upregulated phosphoproteins in M1 FABP5 KO macrophages and 528 downregulated and 393 upregulated phosphoproteins in M2 FABP5 KO macrophages (Fig. 3C, 3D). Importantly, the majority of the differentially regulated proteins and phosphoproteins were distinct from those identified in unstimulated macrophages (Supplemental Fig. 1E, F).

**Figure 4:**
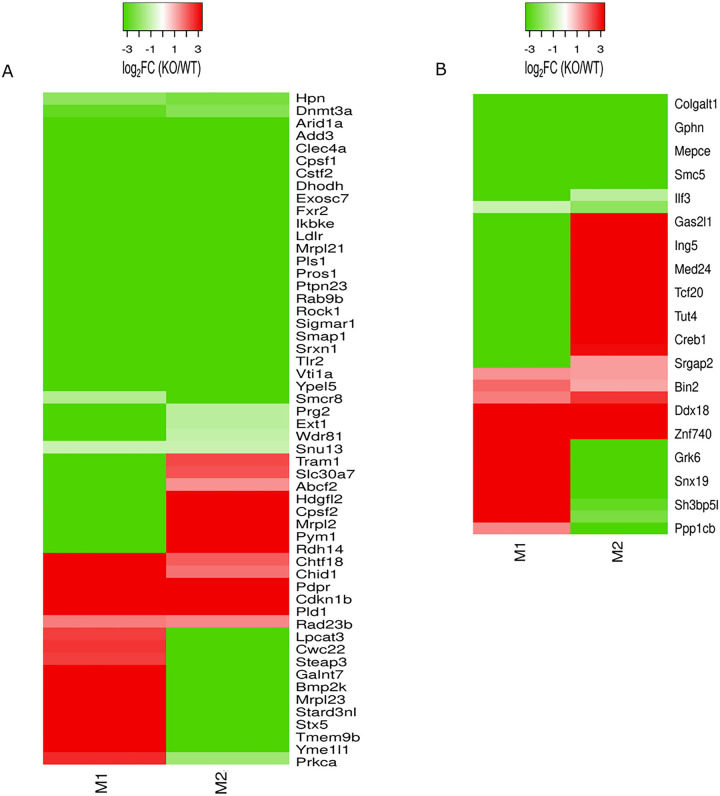
Overlap of proteins and phosphoproteins altered in M1 and M2 BMDMs. Heatmaps depicting differentially regulated proteins **(A)** and phosphoproteins **(B)** altered in both M1 and M2 conditions are shown. The heatmaps represent log_2_FC of FABP5 KO/WT.

We next analyzed differentially regulated proteins and phosphoproteins in WT and FABP5 KO BMDMs that were common to both the M1 and M2 polarized conditions. Shared proteins and phosphoproteins from unstimulated cells were omitted from the analysis. A total of 54 proteins and 37 phosphoproteins were shared between M1 and M2 conditions (Fig. 4, Supplemental Fig. 1E, F). A prominent example is toll-like receptor 2 (Tlr2), which exhibits downregulation in M1 and M2 BMDMs. Tlr2 is linked to inflammation and reactive oxidative species (ROS) production, and its activation enhances the generation of nitric oxide and inflammatory cytokines in macrophages (23). Protein kinase C alpha (Prkca) also displays differential regulation between each polarization condition. Prkca contributes to protection against LPS-induced inflammation in RAW264.7 macrophages as well as ROS production in cancer cell-lines (24, 25), suggesting that FABP5 may modulate ROS levels in macrophages. Upregulation of DEAD-box helicase 18 (Ddx18) and differential regulation of G-protein-coupled receptor kinase 6 (Grk6) were observed between M1 and M2 conditions. Ddx18 exerts a key function in cell-cycle progression while Grk6 modulates inflammation and pathological pain (26–28).

### Proteomic and phosphoproteomic enrichment analysis of M1 and M2 polarized BMDMs

**Figure 5:**
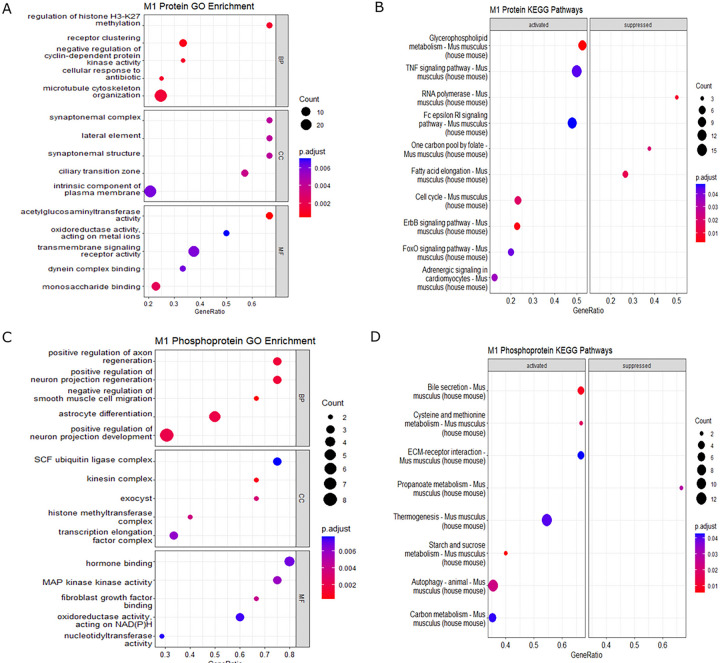
Enrichment analysis of total proteins and phosphoproteins from M1 polarized BMDMs. Protein and phosphoprotein expression from FABP5 KO/WT along with their fold changes were used for enrichment analysis. GO annotation **(A)**and enriched KEGG pathways **(B)** were determined from total protein. GO annotation **(C)** and enriched KEGG pathways **(D)** were identified from all phosphoproteins. Enrichment analyses were performed using a p-value cutoff of 0.05.

We next mapped the biological and cellular functions of the differentially expressed proteins in M1 macrophages. Gene ontology (GO) enrichment revealed processes related to biological process (BP), cellular component (CC), and molecular function (MF). GO enrichment of total proteins identified processes related to receptor clustering, calcium channel complex, and negative regulation of cytokine production (Fig. 5A, Supplemental File S2). KEGG pathway analysis identified differentially expressed pathways including TNF, ErbB, FoxO, and Fc epsilon RI signaling (Fig. 5B, Supplemental File S3). Enrichment analysis for phosphoproteins identified processes including SCF ubiquitin ligase complex, kinesin complex, histone complexes MAP kinase activity, cysteine and methionine metabolism, and ribosome biogenesis (Figs. 5C, 5D).

**Figure 6:**
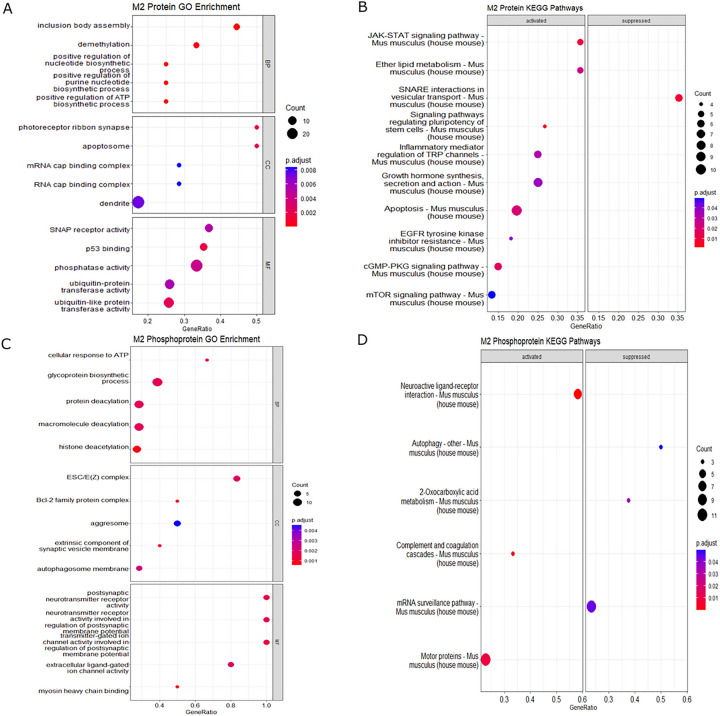
Enrichment analysis of total proteins and phosphoproteins from M2 polarized BMDMs. Protein and phosphoprotein expression from FABP5 KO/WT along with their fold changes were used for enrichment analysis. GO annotation **(A)** and identification of enriched KEGG pathways **(B)** were based on analysis of total proteins. GO annotation **(C)** and enriched KEGG pathways **(D)** were determined from phosphoproteins. Enrichment analyses were performed using a threshold of p < 0.05.

Enrichment analysis of M2 polarized macrophages identified demethylation, regulation of nucleotide biosynthetic process, SNAP receptor activity, and phosphatase activity among differentially expressed pathways (Fig. 6A). Additionally, KEGG analysis highlighted the involvement of JAK-STAT, SNARE interactions, and inflammatory mediator regulation of TRP channels (Fig. 6B). The phosphoprotein dataset demonstrated enrichment in pathways linked to histone deacetylation, Bcl-2 family protein complex, autophagosome membrane, and complement and coagulation cascades (Figs. 6C, 6D). Collectively, these results demonstrate that FABP5 deletion impacts multiple pathways associated with macrophage function and inflammation.

### Kinase enrichment analysis and ROS levels in M1 and M2 polarized BMDMs

**Figure 7:**
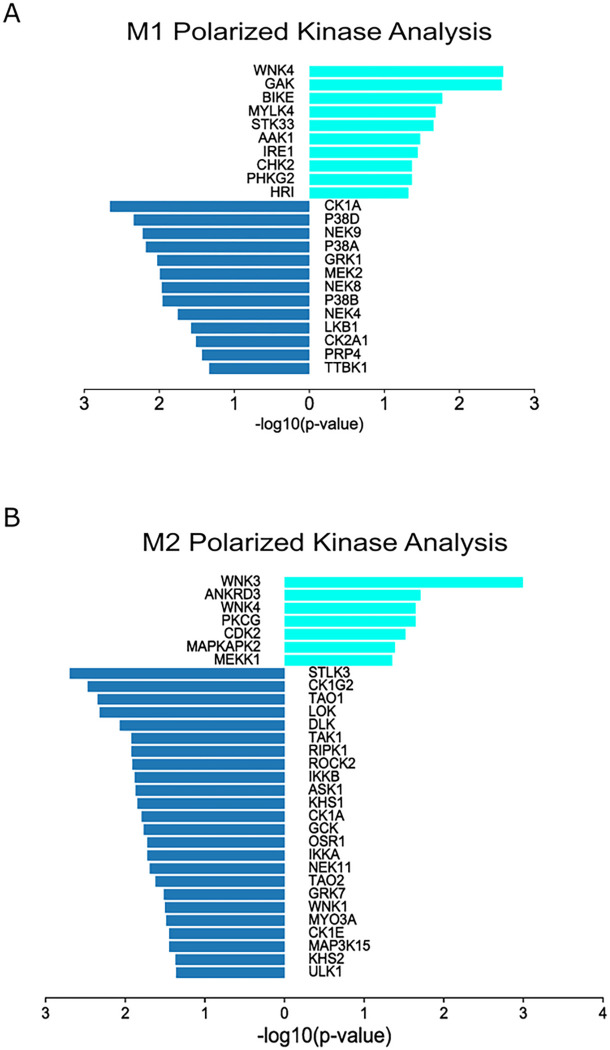
Kinase enrichment analysis of M1 and M2 proteins and phosphoproteins. Kinase enrichment analysis was performed using the phosphorylated sites in M1 **(A)**and M2 **(B)** polarized FABP5 KO/WT BMDMs. Top kinases are depicted, with downregulated kinases shown in dark blue and upregulated kinases shown in light blue.

The kinase-substrate analysis aimed to determine the kinases enriched in FABP5 KO/WT BMDMs under each polarization condition. The enrichment analyses identified terms related to kinases that are enriched in FABP5 KO/WT samples, indicating that the expression of FABP5 and its downstream targets may influence the activity of these kinases. Supplemental Fig. 2 displays a network of enriched kinase, along with their corresponding phosphorylation sites. The analysis identified serine/threonine kinases from the Grk, Prk/Ark, and MapK family in both M1 and M2 (Supplemental Fig. 2). When focusing on PhosphositePlus kinase enrichment based on sequence inputs, similar kinases from these subfamilies were highlighted. Figure 7A depicts the top kinases for M1, with CK1A and GRK1 being downregulated while AAK1 and WNK4 are upregulated. Supplemental Fig. 2B shows that GRK6 is upregulated for M1 polarized samples. GRKs have been increasingly studied for their role in inflammation and chronic inflammatory diseases, such as rheumatoid arthritis, where reduced GRK activity was observed in peripheral blood mononuclear cells (28, 29). Figure 7A and supplemental Fig. 2B also demonstrate that members of the PRK family and WNK family are upregulated for the M1 condition. Notably, WNK4 has been linked to LPS-induced macrophage activation with WNK4 knockout mice showing suppressed inflammation cascade activation (30). For M2, the prominent kinases included CDK being upregulated and MAPK kinases being both up and downregulated (Fig. 7B, Supplemental Fig. 2C and D). It is important to note that GRKs have G-protein independent functions in cell signaling that involve activation of MAPK signaling pathways which are implicated in regulating metabolism, cell survival, cell motility stress response, and inflammation (29, 31).

**Figure 8:**
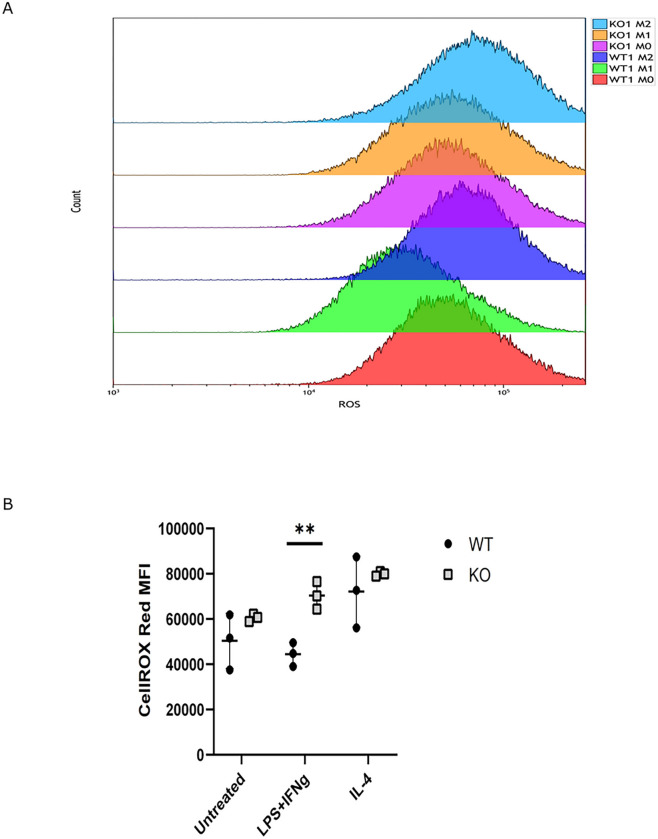
ROS levels in WT and FABP5 KO BMDMs. **(A)** ROS levels in unstimulated as well as M1 and M2 polarized BMDMs were analyzed by flow cytometry with representative results shown. **(B)** ROS levels were quantified in each polarization condition and presented as MFI values. **, p < 0.01.

Interestingly, these kinases are involved in various cellular processes including ROS production (32–35). In addition to the kinase enrichment analysis, our proteomic and phosphoproteomic pathway enrichment results identified several pathways associated with ROS production including TNF pathway, FoxO, and oxidoreductase activity (Fig. 5B, Supplemental File S2, Supplemental File S3). Consequently, we sought to quantify ROS levels in WT and FABP5 KO BMDMs following M1 and M2 polarization. Compared to WT BMDMs, FABP5 deficiency elevated ROS levels in M1 polarized BMDMs (Fig. 8). As ROS are involved in multiple stages of the inflammatory response (36), these results further highlight the potential involvement of FABP5 in regulating macrophage function.

## Discussion

Our study employed SILAC-based quantitative approaches to evaluate global changes in the proteomic and phosphoproteomic landscape of FABP5 KO and WT BMDMs following M1 and M2 polarization. Currently, the role of FABP5 in modulating macrophage inflammatory responses is poorly understood. Previous investigations of pathways modulated by macrophage FABP5 have been largely restricted to the transcriptional level while the current study provides additional insights at the proteomic level. Our investigation revealed key differences in protein expression patterns and downstream pathways between WT and FABP5 KO BMDMs. FABP5 KO BMDMs were marked by differential expression of hundreds of proteins that mapped to distinct downstream pathways including oxidoreductase activity, MAP kinase activity, JAK-STAT signaling pathway, TNF signaling pathway, and multiple others. These pathways collectively contribute to key functions including the regulation of proliferation, survival, inflammatory and immune responses, receptor-mediated activation, and cellular signaling. Our findings largely align with prior transcriptomic studies performed in WT and FABP5 KO BMDMs. For example, transcriptomics revealed that pathways related to TNF signaling and Fc gamma R-mediated phagocytosis are enriched in M1 polarized FABP5 KO BMDMs (14). KEGG analysis identified activation of the TNF signaling pathway but also Fc epsilon RI receptor mediated signaling in FABP5 KO BMDMs (Fig. 5B). Fc epsilon RI and Fc gamma R both recognize the Fc portion of antibodies and activate intracellular signaling cascades upon ligand binding (37). FcεRI signaling contributes to the differentiation of monocytes to pro-inflammatory histamine receptor 1-expressing macrophages (38, 39). Our results also identified differences with previous findings. For example, a transcriptomic analysis found elevated expression of NFkB2 in untreated FABP5 KO BMDMs (40), which was not reflected in our study. Such results further attest to the importance of conducting parallel transcriptomic and proteomic analyses. Overall, the pathway analyses highlight the likely involvement of FABP5 in modulating macrophage inflammatory responses.

Recent studies have implicated FABP5 in the regulation of ROS production via NADPH oxidase (NOX) (41). FABP5 deficiency reprograms metabolic pathways leading to increased ATP production and reduction in ROS biosynthesis (14). However, another investigation reported that spleen monocytes derived from FABP5 KO mice exhibit elevated levels of ROS, and KO BMDMs secrete increased levels of nitrate anion (42). Interestingly, we found that FABP5 deficiency enhanced cellular ROS levels in M1 polarized BMDMs. These discordant findings may stem from methodological differences between studies including differential polarization conditions and time courses for ROS quantification. For example, a 5 min stimulation of WT BMDMs with LPS increases ROS production while our study did not reveal an increase in ROS levels following stimulation for 24h (41). It is noteworthy that GRK family kinases were highly represented throughout our pathway and phosphoproteomic analyses. GRK6 regulates ROS responses in T cells (43) and may exert a similar function in macrophages.

One limitation of the current study is the focus on BMDMs as it is possible that FABP5 may regulate distinct transcriptomic, proteomic, and phosphoproteomic responses in tissue resident or tumor-associated macrophages. Another limitation stems from the use of cells bearing a constitutive deletion of FABP5. It is currently not known whether transient pharmacological FABP5 inhibition produces overlapping responses compared to those observed in FABP5 KO cells. Given the established and emerging functions of macrophages and FABP5 in inflammatory processes and pain (44–46), future studies will be required to clarify the functions of macrophage FABP5 in a myriad of physiological and pathophysiological processes.

## Conclusions

This is the first study to profile the impact of FABP5 deficiency upon the proteomic and phosphoproteomic landscape of M1 and M2 polarized BMDMs. Loss of FABP5 was characterized by upregulation and downregulation of distinct pathways associated with macrophage inflammatory responses. These findings establish a framework for future efforts aimed at exploring the therapeutic potential of FABP5 in inflammatory processes.

## Data Availability

All raw datasets are available in the MassIVE protein data repository (massive.ucsd.edu) and the DRYAD database. The GO and KEGG datasets are available as supplementary files. Any other datasets analyzed for this study are available from the corresponding author upon reasonable request.
